# Supplementary figures and images for: Reassessment of the involvement of Snord115 in the serotonin 2c receptor pathway in a genetically relevant mouse model
Source: eLife. 2020 Oct 5;9:e60862. doi: 10.7554/eLife.60862 (PMC7673782; doi:10.7554/eLife.60862)

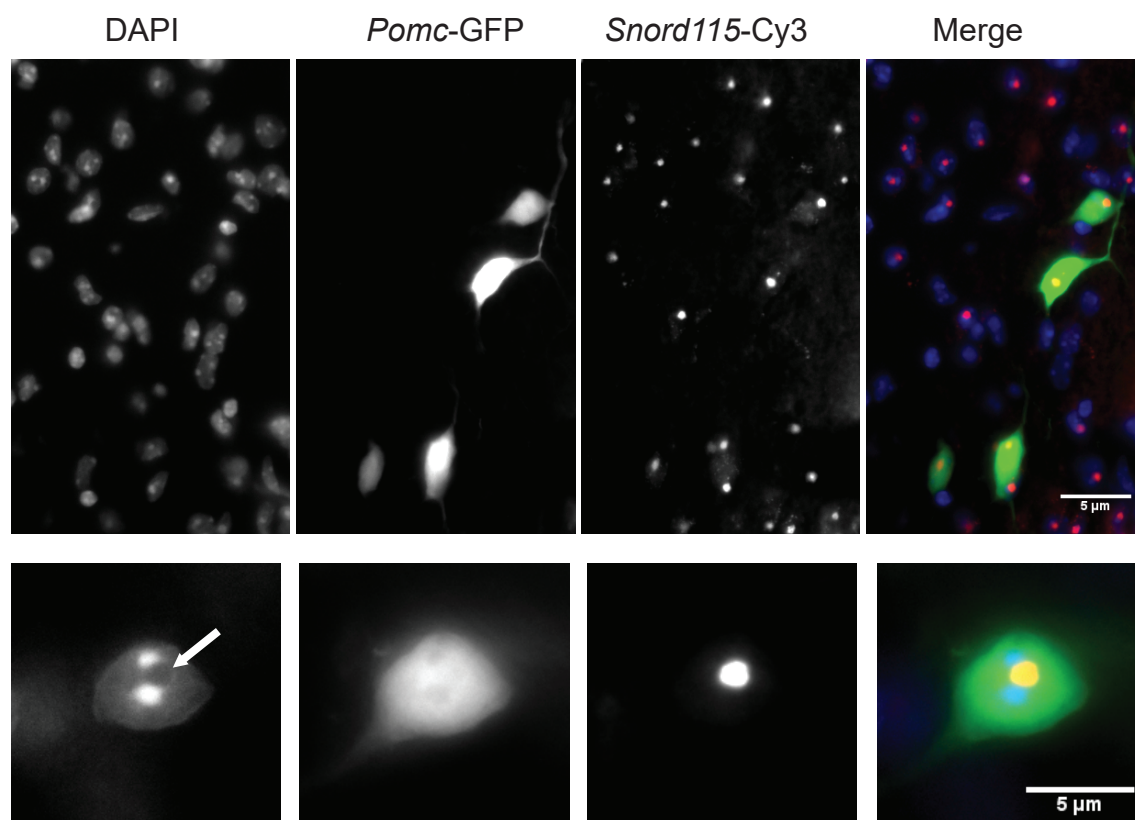

Supplement: Supplementary file 2. — Coronally sectioned brain tissues of adult Pomc-eGFP transgenic mice (Pomc neurons appear green in the merge) were hybridized with Cy3-labeled DNA oligonucleotides (red signals in the merge). Bottom: Snord115 signals are detected in nuclear regions poorly stained by DAPI (white arrow), very likely representing nucleoli. [file elife-60862-supp2.pdf]
