# Supplementary material for: Reassessment of the involvement of Snord115 in the serotonin 2c receptor pathway in a genetically relevant mouse model
Source: eLife. 2020 Oct 5;9:e60862. doi: 10.7554/eLife.60862 (PMC7673782; doi:10.7554/eLife.60862)
Supplement: Transparent reporting form [file elife-60862-transrepform.docx]

***eLife’s* transparent reporting form**

We encourage authors to provide detailed information *within their submission* to facilitate the interpretation and replication of experiments. Authors can upload supporting documentation to indicate the use of appropriate reporting guidelines for health-related research (see [EQUATOR Network](http://www.equator-network.org/%20)), life science research (see the [BioSharing Information Resource](https://biosharing.org/)), or the [ARRIVE guidelines](http://www.plosbiology.org/article/info:doi/10.1371/journal.pbio.1000412) for reporting work involving animal research. Where applicable, authors should refer to any relevant reporting standards documents in this form.

If you have any questions, please consult our Journal Policies and/or contact us: [editorial@elifesciences.org](mailto:editorial@elifesciences.org).

**Sample-size estimation**

- You should state whether an appropriate sample size was computed when the study was being designed
- You should state the statistical method of sample size computation and any required assumptions
- If no explicit power analysis was used, you should describe how you decided what sample (replicate) size (number) to use

Please outline where this information can be found within the submission (e.g., sections or figure legends), or explain why this information doesn’t apply to your submission:

**The phenotypic exploration described in this study is common in mice, notably within the C57BL/6J genetic background. The number of analyzed individuals was mostly inferred based on our own experience and that of the “Mouse Behavioral Core» at the Center of Integrative Biology (behavioral studies) or the ANEXPLO platform at the Institute of Cardiovascular and Metabolic Diseases (metabolic studies) in Toulouse. Sample sizes of sufficient power were also chosen on the basis of similar published research and taking into consideration the 3 Rs principles (Replacement-Reduction-Refinement) that govern the ethics of animal use in research. The numbers of replicates/samples are indicated, either in the Figure Legend or below the histograms.**

**Replicates**

- You should report how often each experiment was performed
- You should include a definition of biological versus technical replication
- The data obtained should be provided and sufficient information should be provided to indicate the number of independent biological and/or technical replicates
- If you encountered any outliers, you should describe how these were handled
- Criteria for exclusion/inclusion of data should be clearly stated
- High-throughput sequence data should be uploaded before submission, with a private link for reviewers provided (these are available from both GEO and ArrayExpress)

Please outline where this information can be found within the submission (e.g., sections or figure legends), or explain why this information doesn’t apply to your submission:

**In our study, biological replicates mean individual mouse or RNA samples prepared from one individual tissue. We compared mutant mice with their wild-type littermates by crossing a heterozygous male with a wild-type female.** **Depending on Mendelian and sex ratios, a cohort (8-13 individuals per genotype) was obtained from 4-8 independent litters.**

- **Behavioral and metabolic phenotyping - The exact group size (n) for each experimental group/condition is provided in the Figure legends, and ‘n’ refers to independent values, not replicates.**
- **Outliers Figure 4 - Behavioural studies were carried out using two different cohorts**

**Cohort #1 (Fig4A-E): 12WT vs 13KO (OF 🡺EPM🡺TST🡺FST🡺NSF)**

**EPM - Three mice could not be analysed (2WT/1KO) because the mice fell off the device.**

**NSF – Three mice could not be analysed (2WT/1KO) due to uncertainty on identification and/or scoring issues.**

**Cohort #2 (Fig4F): 9WT vs 8KO (social tests)**

**A WT mouse was excluded from analysis (left panel) due to technical issues during recording.**

- **mCPP treatment - Each genotype was first treated with a saline solution (NaCl, control) and then with mCPP (1 mg/kg or 5 mg/kg). Both injections were performed at 10-14 days interval.**
- **Analyses of A-to-I RNA editing - Raw data are available on Sequence Read Archive (SRA) database under the accession numbers PRJNA603261 and PRJNA603264. Scripts used for that analysis, detailed instructions and intermediary data have been deposited at** [**https://github.com/HKeyHKey/Hebras_et_al_2020**](https://github.com/HKeyHKey/Hebras_et_al_2020)**.**

**For SRA dataset PRJNA603261 (various adult brain areas, in SNORD115-expressing and SNORD115-deficient specimens; 202 libraries):** [**https://dataview.ncbi.nlm.nih.gov/object/PRJNA603261?reviewer=o3er4up2m957p1n2i0q1ndicp7**](https://dataview.ncbi.nlm.nih.gov/object/PRJNA603261?reviewer=o3er4up2m957p1n2i0q1ndicp7)

**For SRA dataset PRJNA603264 (various embryonic, neonate and adult brain areas, in SNORD115-expressing and SNORD115-deficient specimens; 191 libraries): https://dataview.ncbi.nlm.nih.gov/object/PRJNA603264?reviewer=chjkrf27geapfsqlif4rs3t2u9**

- **mRNA-seq - Raw data are available on Sequence Read Archive (SRA) database under the accession number PRJNA608249.**

[**https://dataview.ncbi.nlm.nih.gov/object/PRJNA608249?reviewer=a7t5r3isdi140u3hb8f4i4ur7t**](https://dataview.ncbi.nlm.nih.gov/object/PRJNA608249?reviewer=a7t5r3isdi140u3hb8f4i4ur7t)

- **RiboMeth-seq - Raw data are available on GEO under the accession number GSE145159. Secure token is: qpmfwmqeppyzhev**

**Statistical reporting**

- Statistical analysis methods should be described and justified
- Raw data should be presented in figures whenever informative to do so (typically when N per group is less than 10)
- For each experiment, you should identify the statistical tests used, exact values of N, definitions of center, methods of multiple test correction, and dispersion and precision measures (e.g., mean, median, SD, SEM, confidence intervals; and, for the major substantive results, a measure of effect size (e.g., Pearson's r, Cohen's d)
- Report exact p-values wherever possible alongside the summary statistics and 95% confidence intervals. These should be reported for all key questions and not only when the p-value is less than 0.05.

Please outline where this information can be found within the submission (e.g., sections or figure legends), or explain why this information doesn’t apply to your submission:

**Dispersion and precision measures (mean, SEM), statistical tests used as well as the number of replicates (n) and p-values are given for each figure, or in the Method section. Statistical analysis methods are given in the Method section. Data files and R scripts for these analyses have been deposited on** [**https://github.com/HkeyHKey/Hebras_et_al_2020**](https://github.com/HkeyHKey/Hebras_et_al_2020) **and**

(For large datasets, or papers with a very large number of statistical tests, you may upload a single table file with tests, Ns, etc., with reference to sections in the manuscript.)

**Group allocation**

- Indicate how samples were allocated into experimental groups (in the case of clinical studies, please specify allocation to treatment method); if randomization was used, please also state if restricted randomization was applied
- Indicate if masking was used during group allocation, data collection and/or data analysis

Please outline where this information can be found within the submission (e.g., sections or figure legends), or explain why this information doesn’t apply to your submission:

**Age- and sex-matched cohorts of SNORD115-KO and WT littermates were compared. As stated in the Method section, most behavioral tests, metabolic studies and pharmacological treatments were performed blind during the light phase (from 8:30 a.m to 1 p.m.) using 3- to 5- month-old male mice. In order to limit potential confounding effects due to genetic heterogeneity and/or undesired CRISPR-Cas9-mediated cleavages, if any, we backcrossed to C57BL/6J background for at least 8 generations before proceeding to phenotypic exploration.** **Note that where possible, we sought to randomize animals**

**Additional data files (“source data”)**

- We encourage you to upload relevant additional data files, such as numerical data that are represented as a graph in a figure, or as a summary table
- Where provided, these should be in the most useful format, and they can be uploaded as “Source data” files linked to a main figure or table
- Include model definition files including the full list of parameters used
- Include code used for data analysis (e.g., R, MatLab)
- Avoid stating that data files are “available upon request”

Please indicate the figures or tables for which source data files have been provided:

- **Figure 1G (supplementary data file 1; RiboMeth-seq)**
- **Figures 3A-3B (**[**https://github.com/HKeyHKey/Hebras_et_al_2020**](https://github.com/HKeyHKey/Hebras_et_al_2020)**)**
- **Figure 5M (supplementary data file 3 ; mRNA-seq)**
- **List of primers (supplementary data file 4)**

**R and the Graphpad Prism® (version 8) statistical software were used for data analysis.**
